# The Acquisition of Noun and Verb Categories by Bootstrapping From a Few Known Words: A Computational Model

**DOI:** 10.3389/fpsyg.2021.661479

**Published:** 2021-08-19

**Authors:** Perrine Brusini, Olga Seminck, Pascal Amsili, Anne Christophe

**Affiliations:** ^1^Department of Psychological Sciences, University of Liverpool, Liverpool, United Kingdom; ^2^Laboratoire de Sciences Cognitives et Psycholinguistique, Centre National de la Recherche Scientifique, École Normale Supérieure/PSL University, Paris, France; ^3^Laboratoire Langues, Textes, Traitements Informatiques, Cognition (Lattice), Centre National de la Recherche Scientifique, École Normale Supérieure/PSL University, Université Sorbonne Nouvelle, Paris, France

**Keywords:** language development, acquisition of syntax, computational modeling, semantic seed, noun, verb, French

## Abstract

While many studies have shown that toddlers are able to detect syntactic regularities in speech, the learning mechanism allowing them to do this is still largely unclear. In this article, we use computational modeling to assess the plausibility of a context-based learning mechanism for the acquisition of nouns and verbs. We hypothesize that infants can assign basic semantic features, such as “is-an-object” and/or “is-an-action,” to the very first words they learn, then use these words, the *semantic seed*, to ground proto-categories of nouns and verbs. The contexts in which these words occur, would then be exploited to bootstrap the noun and verb categories: unknown words are attributed to the class that has been observed most frequently in the corresponding context. To test our hypothesis, we designed a series of computational experiments which used French corpora of child-directed speech and different sizes of semantic seed. We partitioned these corpora in training and test sets: the model extracted the two-word contexts of the seed from the training sets, then used them to predict the syntactic category of content words from the test sets. This very simple algorithm demonstrated to be highly efficient in a categorization task: even the smallest semantic seed (only 8 nouns and 1 verb known) yields a very high precision (~90% of new nouns; ~80% of new verbs). Recall, in contrast, was low for small seeds, and increased with the seed size. Interestingly, we observed that the contexts used most often by the model featured function words, which is in line with what we know about infants' language development. Crucially, for the learning method we evaluated here, all initialization hypotheses are plausible and fit the developmental literature (semantic seed and ability to analyse contexts). While this experiment cannot prove that this learning mechanism is indeed used by infants, it demonstrates the feasibility of a realistic learning hypothesis, by using an algorithm that relies on very little computational and memory resources. Altogether, this supports the idea that a probabilistic, context-based mechanism can be very efficient for the acquisition of syntactic categories in infants.

## Introduction

In the past decades, many experimental studies have shown that young children start gathering knowledge about the syntactic structure of their native language much earlier than was initially thought. For instance, infants are sensitive to the function words of their language before their first birthday (e.g., Shafer et al., 1998; Shi et al., 2006a; Halle et al., 2008), and they start exploiting them to speed up their lexical access to already acquired content words between 12 and 18 months (e.g., in English or French, determiners are followed by nouns, personal pronouns by verbs, Kedar et al., 2006, 2017; Zangl and Fernald, 2007; van Heugten and Johnson, 2011; Cauvet et al., 2014). In addition, when presented with novel content words in several contexts, infants are able to infer which other contexts are expected for these novel words: for instance, after hearing *the blick*, then *a blick* would be expected but not *I blick* (for German: Höhle et al., 2004; for French: Shi and Melançon, 2010). Starting at 12–14 months of age, toddlers can exploit the syntactic contexts of novel content words to infer their plausible meaning—for instance, a novel word presented in a noun context, such as *it is a blick*, is assumed to refer to an object (e.g., Waxman, 1999; Waxman and Booth, 2001), while it is assumed to refer to an action if it is heard in a verb context, such as *he's blicking* (from 18 months on, Bernal et al., 2007; Waxman et al., 2009; Oshima-Takane et al., 2011; He and Lidz, 2017; de Carvalho et al., 2019). Around 20 months, toddlers also start to exploit the syntactic structure in which novel verbs appear to constrain their possible meaning—specifically mapping verbs appearing in transitive structures to causal actions (Yuan and Fisher, 2009; Arunachalam and Waxman, 2010; Fisher et al., 2010; Dautriche et al., 2014; de Carvalho et al., 2021).

These studies have established that the syntactic structure in which a word appears is exploited by toddlers to guess some of the probable characteristics of its referent. Depending on their syntactic contexts, words are attributed plausible semantic features, such that for instance, nouns are considered likely to refer to objects, and verbs likely to refer to actions (and similarly for different kinds of actions, such as 1-participant vs. 2-participants actions, and properties for adjectives). This wealth of experimental research was triggered by the *syntactic bootstrapping* hypothesis proposed by Lila Gleitman in the 80s (Landau and Gleitman, 1985; Gleitman, 1990), stating that very young children could exploit syntactic structure to constrain their learning of word meanings, by relying on the link between grammatical form and semantic characteristics (see also Waxman and Hall, 1993; Fisher et al., 1994; Fisher, 1996 and the excellent discussion in Waxman and Lidz, 2006). Since then, many studies have successfully demonstrated that some syntactic knowledge is available to children early in development, when they still have a fairly limited lexical knowledge. However, all these experimental results raise the question of *how* toddlers manage to figure out which contexts correspond to specific syntactic categories.

One possibility is that infants are able to analyze the distributional information of their input to identify words which occur in the same contexts as words from specific categories (Redington et al., 1998; Seidenberg and MacDonald, 1999). Several unsupervised computational models used the local context of words to assign them a category (Redington et al., 1998; Mintz, 2003; Parisien et al., 2008; Chemla et al., 2009; Chrupała and Alishahi, 2010; Weisleder and Waxman, 2010; Wang et al., 2011). They all presented better-than-chance performance in a categorization task, showing that local contexts do indeed contain relevant information. Because these models are unsupervised, they present the advantage that they pre-suppose no specific linguistic knowledge from infants. However, they run into several difficulties, that vary depending on the implementation choices that were made. For instance, Redington et al.'s model attempts categorization only for words which have been observed very often (the 1,000 most frequent words of the corpus), and groups words together based on the similarity of the contexts they occur in. Because it possesses very rich information regarding all the contexts that each to-be-categorized word may enter, it outputs a rich and accurate set of categories, for both content and function words (which are much represented in the 1,000 most frequent words). However, because this model does not even attempt categorization for new words or the ones that are seen only a few times, it is not particularly useful to describe how toddlers constrain word meaning acquisition, since these are precisely the words where additional information would come in handy to guess their meaning.

Other models have focused on frequent contexts rather than frequent to-be-categorized words, with the advantage that these models can categorize even words that are seen for the first time. In these models, the clustering mechanisms typically yield many different classes, with several classes for each target linguistic category (Mintz, 2003; Chemla et al., 2009; Gutman et al., 2015). For instance, in the “frequent frames” framework developed by Mintz (2003), the model starts by identifying the pairs of words that co-occur most frequently, with a gap of 1 word in-between. It turns out that words that are sandwiched within these contexts of frequently co-occurring words tend to share their category: for instance, *you _ it* selects verbs, while *the _ is* selects nouns. The end result of this procedure returns several groups of word for each syntactic category; for instance, there are several noun classes, corresponding to the frames *the _ is*, and *a _ is*, among others. Attempts to group classes together on the basis of shared words are not trivial, because many words belong to more than one category (e.g., noun/verb, “I bear,” “the bear”). In an attempt to escape the tension between categorizing only a restricted number of frequent words and building many classes for the same categories, we present a model that is trained on a corpus in which a few words are initially categorized: the *semantic seed*.

The semantic seed refers to a plausible assumption: by the time children start addressing the categorization problem, they already have managed to learn the meaning of a few highly frequent content words. In addition, we hypothesized that infants are able to group those known words according to some semantic feature (e.g., words referring to objects, words referring to actions). Findings from the literature make both parts of this hypothesis highly plausible. First, several studies have shown that infants have already built a small lexicon before their first birthday (Bergelson and Swingley, 2012, 2013, 2015; Parise and Csibra, 2012; Syrnyk and Meints, 2017). For instance, Bergelson and Swingley (2012, 2013, 2015) have shown that 6- and 9-month-old babies already know some nouns and some verbs. This demonstrates that word learning can occur very early, even when infants have very little linguistic knowledge yet. In some situations, the non-linguistic context is sufficiently supportive to promote word learning: namely when words have clear, concrete referents (objects and actions in the here and now, Medina et al., 2011; Taxitari et al., 2020), and when the context of the conversation contains rich socio-pragmatic cues (Tomasello and Akhtar, 1995; Akhtar et al., 1996). Second, it has been proposed that infants are able to detect specific semantic features in their environment and group them to form semantic categories such as agents, artifacts, or actions (Saxe et al., 2006; Carey, 2009). In addition, infants' ability to form categories is enhanced by speech, such that speech sounds seem to promote the formation of an object category in infants (Ferry et al., 2010, 2013), and labeling two objects with different words allows 9-month-old infants to consider them as different kinds (Xu, 2002, see Ferguson and Waxman, 2017 for a review). Other studies focusing on how language encodes some semantic features, such as gender, animacy and number, demonstrated that when semantic attributes are encoded in language, this is learned by infants (Berko, 1958; van Heugten and Shi, 2009; Shi, 2014; Lukyanenko and Fisher, 2016; Ferry et al., 2020). In fact, the range of semantic attributes that are morphosyntactically encoded in languages has been hypothesized to be part of what has been called the *core knowledge* system (Spelke, 2000; Strickland, 2017).

In the present work, we marked the different words known by the model, the semantic seed, as either action-referring words to form the seed of the “verb” category, or object-referring words to form the seed of the “noun” category. This is supported by a body of work showing that toddlers differentiate actions and objects and tend to map the first on verb items and the latter on noun items (Bernal et al., 2007; Waxman et al., 2009; Oshima-Takane et al., 2011; He and Lidz, 2017; de Carvalho et al., 2019). For instance, let's assume that a given infant managed to learn the meaning of “book,” “teddy,” “eat,” “banana,” “go,” and “drink,” (because they are highly frequent and refer to concrete objects and actions), they may be able to group them into [book, banana, teddy]_object referents_ and [go, eat, drink]_action referents_. Starting from this seed, infants would then need a learning mechanism that extends those proto-categories, relying for example on information from their context. By noticing in which contexts the object referents often appear (e.g., after “*and the*,” or “*like a*”), children might be able to decide that an unknown word, such as “*bunny*” in “*and the bunny jumped*,” also belongs to the object-referents category. The model we present here precisely attempts to test the efficacy of such a process.

The model stores two-word contexts for each word from a training corpus, in which a few words are categorized (the *semantic seed*). It then uses these contexts to categorize words in an unseen test corpus. We report here a series of experiments, in which we present the performance of this learning mechanism. We consider different sets of parameters, namely different sizes of the semantic seed and three different types of two-word-sized contexts: left, right and framing contexts. Evaluation of the model was obtained by carrying out a categorization task targeting unknown words. To study the impact of the size of the vocabulary known initially, we varied parametrically the size of the semantic seed (starting with only a handful of known words, up to a much more sizeable vocabulary).

To sum up, the aim of this study is to conduct a feasibility experiment and check how much knowledge infants could gather about the noun and verb categories, if they had access to the kind of computation hypothesized by the model. The model rests on two main assumptions which are both plausible and grounded in the infant literature. First, the *semantic seed* assumption proposes that when they approach the categorization task, infants have already succeeded in learning the meaning of a few words (frequent, referring to concrete objects and actions, presented in pragmatically helpful situations), and are able to group them into semantic classes: object referents and action referents (both parts of the assumption well supported by the infant literature, as seen above). Second, the model supposes that infants are able to keep track of bi- and trigram frequencies: a number of experiments support this assumption, showing that infants as young as 12 months pay attention to this type of distributional information, both when exposed to artificial languages (e.g., Gomez and Gerken, 1999; Marchetto and Bonatti, 2013), or when listening to sentences in their mother tongue (e.g., Santelmann and Jusczyk, 1998; Höhle et al., 2006; van Heugten and Johnson, 2010; van Heugten and Christophe, 2015). Note that the model is mimicking comprehension, since it attempts to categorize words from its input (on the basis of their linguistic context), in the hope of guessing their potential meanings, just as an infant would do when attempting to decode language.

In addition to these assumptions, the model has another important property: It categorizes words only in context. In other words, the model's main aim is not to produce a lexicon in which each word is listed together with its category—or, in the (rather frequent) case of words with more than one category, with its possible categories. Instead, each to-be-categorized word is classified as a function of its immediate context, irrespective of the nature of the word itself. Because of this characteristic, the model can classify words that are encountered for the first time (a useful feature if categorization is going to help word meaning acquisition) and should not suffer when it encounters an ambiguous word.

## Materials and Methods

Our model is based on a corpus of child-directed speech and keeps track of the frequency of triplets of adjacent words. It starts out knowing the categories of a few content words, that are grouped into semantic classes: object-referring and action-referring. At test, the model attempts to categorize some target words by looking at their two words of context. The model targets words that are not too frequent (namely, below a given frequency threshold), since frequent words are less likely to be unknown. As a consequence, the model will mostly target content words, since highly frequent words tend to be function words (for instance, upon hearing the string of words *the door*, one may expect to next find a verb, as in *the door creaks*; if, however the next word is *of*, as in *the door of the house*, the model will not attempt to categorize *of* because it is so frequent). The highly frequent words are, however, used as contexts.

To investigate the impact of the position of the words of context relative to the to-be-categorized word, three different contexts are implemented in three different models: two words immediately preceding the target word—left context; two words immediately following—right context; or one word before and one after—framing context. If these two words belong to trigrams that were observed during training, the model picks as its response the most frequent item occurring with these two words of context. We compare these three contexts to a baseline model: a model that does not rely on context to predict the syntactic category of low frequency words but that randomly predicts “noun,” “verb,” or “other” pondered by the percentage of known nouns and verbs from the corpus.

**Figure 1:**
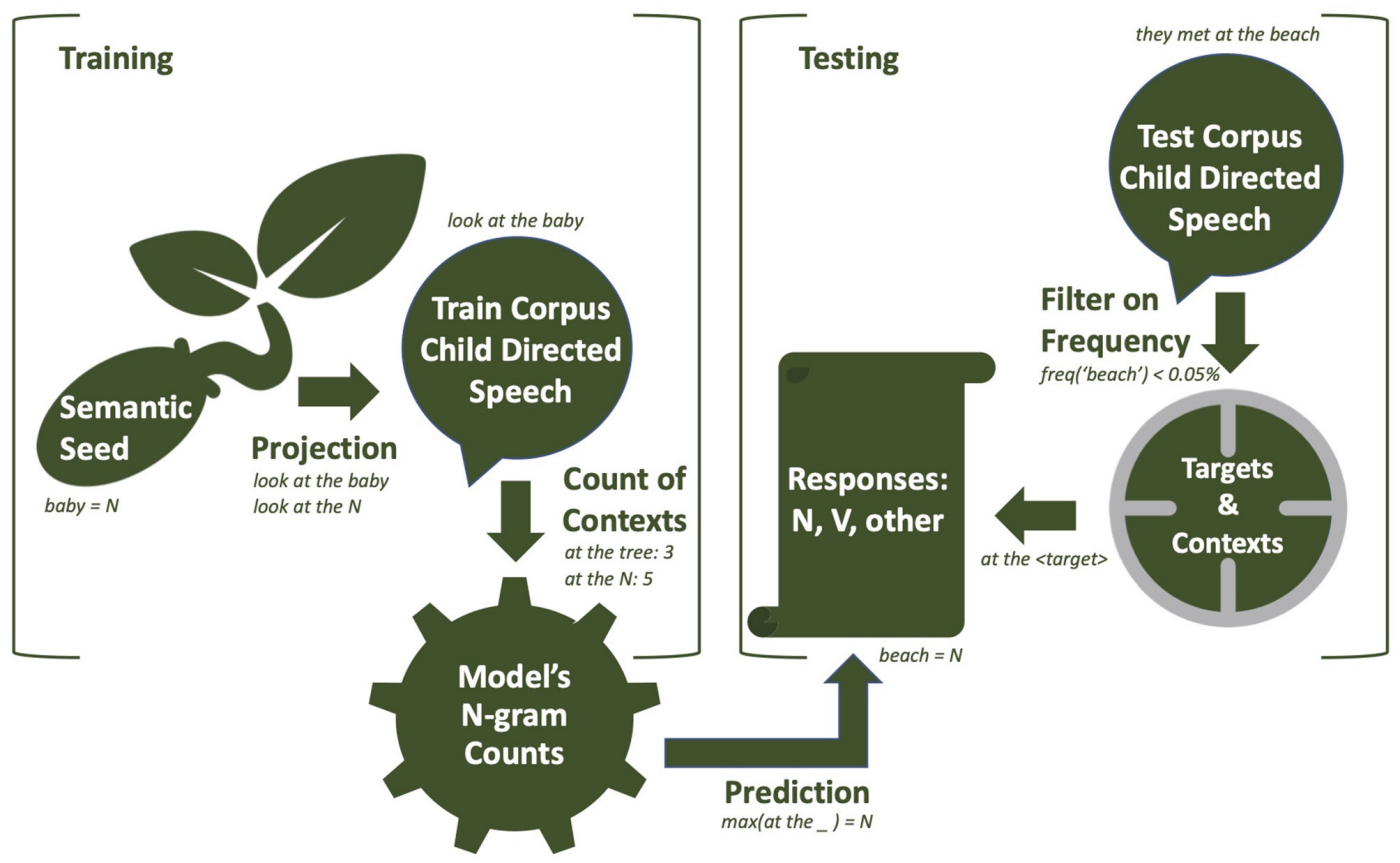
A representation of the different steps of training and testing from our model. Details about the mechanisms can be found in the section Training and Testing.

In this section, we present the details about the model's implementation. In Figure 1, the whole pipeline of our experiment is illustrated by a flow chart. The corpora and scripts are available in a GitHub repository, with the following link: https://github.com/oseminck/bootstrapping_model.

### Corpus

The corpus is a transcription of spontaneous speech produced by French mothers during several play sessions with their child, and available in the CHILDES database (MacWhinney, 2000). The model used transcriptions of two mother/child pairs from the Lyon corpus (available at http://childes.psy.cmu.edu/data/Romance/French/Lyon.zip), Marie and Theotime, aged between 17 and 30 months during the recordings (Demuth and Tremblay, 2008).

The speech produced by the mothers of Marie and Theotime was extracted from the corpus, for a total of 58,241 utterances (265 K tokens). Each word of the corpus was then assigned a category (Part-of-Speech, or POS-tag) to evaluate the model's responses (by comparing the category predicted by the model with the actual category of the word). For the POS-tagging, we used the disambiguation grammar POST developed by Christophe Parisse that is integrated in the CLAN software (the program developed to exploit the CHILDES corpus; MacWhinney, 2000). We merged different types of noun categories and verb categories together (for example, we included modal verbs into the broader category of “verbs”). We performed a manual evaluation of the 640 first tokens of the corpus and found that 9% of the tokens were tagged with the wrong POS-tag. Because we are particularly interested in nouns and verbs, we also evaluated the error rate for these categories. The error rate of tokens tagged as verbs was 0%, but for nouns it was very high: 19%, meaning that 19% of the words that were tagged as nouns did not belong to that category. We therefore applied a correction to the tokens tagged as nouns in the following manner: we extracted all the noun lexemes from the corpus and sorted them by frequency. We then manually judged the 834 most frequent nouns (7 occurrences or more). We selected the lexemes that we suspected not to be nouns. For example, we found the word “*pour*” (the preposition “*for*” in French) in this list. This resulted in a list of 112 suspected lexemes. We then checked in the corpus whether the use was indeed non-nominal and not ambiguous between nouns and another syntactic category. For example, “*pour”* was never a noun, but “*touche”* (to touch/a button) was ambiguous between noun and verb. 100 lexemes were unambiguously non-nominal. We then corrected all the unambiguous lexemes in the corpus, which resulted in 6911 tokens being retagged. The list of the suspected lexemes and the corrected lexemes can be found in the additional materials of this article as well as in the GitHub repository.

### Projection

To implement the idea that a small number of words are already correctly categorized by the learner, we placed an incomplete tier of categories on top of the tier of tokens in the training corpus. We call this tier of POS-tags the projection of the corpus. The category of all the words that belong to the semantic seed are identified in this tier.

### Selection of the Semantic Seed

**Figure 2:**
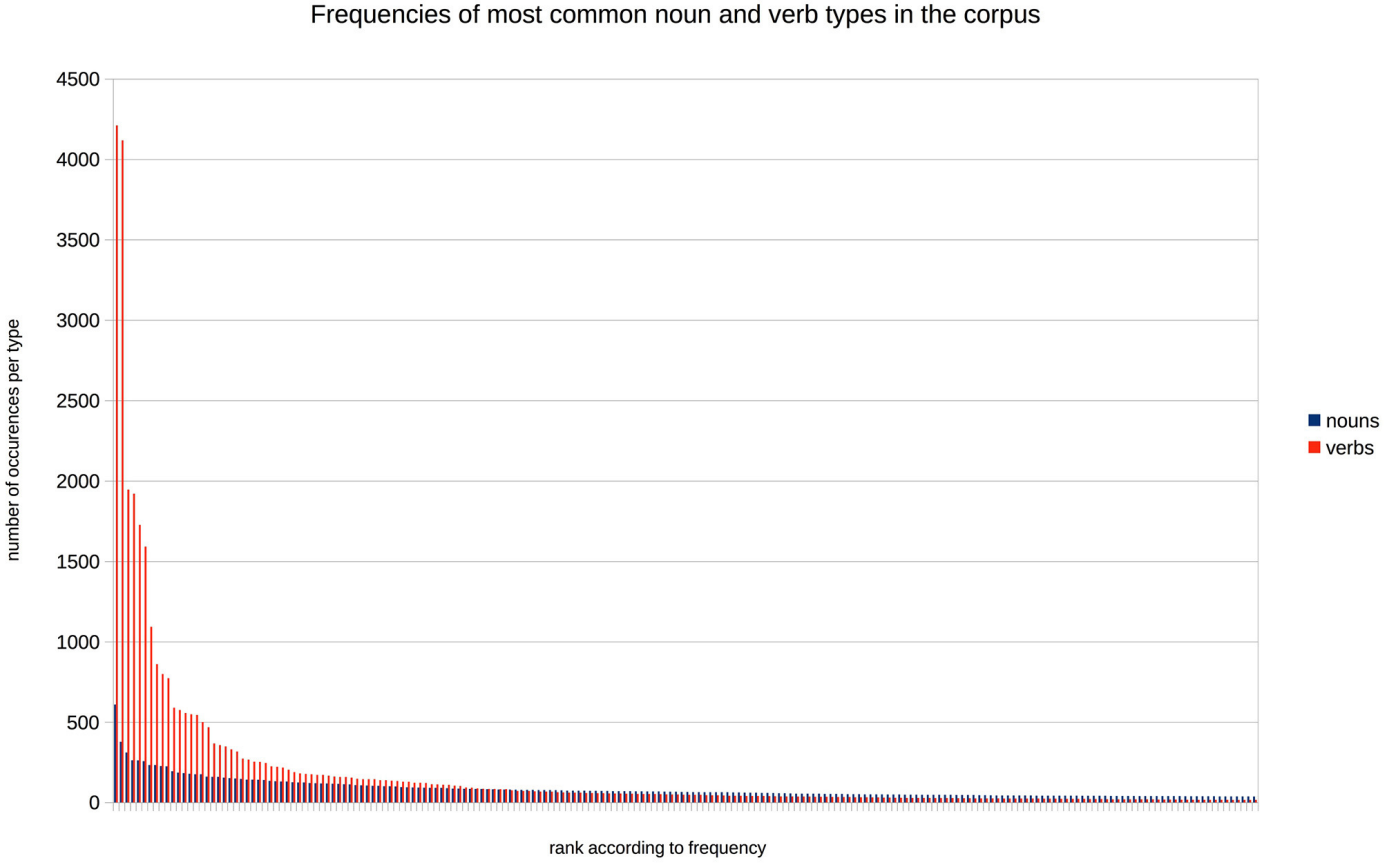
The number of occurrences of the 200 most frequent noun and verb types in the corpus, ranked by frequency.

The semantic seed is composed of the most frequent nouns and verbs from the corpus that respectively refer to objects (including animate entities) and actions. The list of these words is given in Table 1. We varied parametrically the size of the semantic seed, so as to study the impact of the number of tokens initially categorized. As a starting point, we selected a situation in which the learner knows initially only very few of the verb and noun tokens: this corresponds to 8 nouns (7.1% of the noun tokens) and 1 verb (10% of the verb tokens). We then constructed 4 larger vocabulary sets, doubling the number of known nouns at each step, and adjusting the number of verbs such that the percentages of projected noun and verb tokens were relatively similar (increasing the percentage of the projection with about 5–10% for each new semantic seed, see Table 1). The reason why the number of verbs in the smaller semantic seeds is so low, is that these verbs are highly frequent, much more so than the most frequent nouns (see Figure 2)^1^. As a comparison point, one last set of vocabulary was created, containing all the nouns and verbs present in the training corpus (2,159 nouns and 860 verbs). This last vocabulary is obviously not a plausible representation of the lexical knowledge of a toddler, but it gives us an estimate of the best possible performance of the models we are implementing.

**Table 1:** Words of the semantic seeds of various sizes.

	**Nb noun lexemes in semantic seed**	**Percentage of projected nouns**	**Noun lexemes**	**Nb verb lexemes in semantic seed**	**Percentage of projected verbs**	**Verb lexemes**
V0	8	7.3%	bébé, livre, doudou, main, tête, eau, voiture, pied	1	10.9%	aller
V1	16	11.8%	V0 + micro, nez, maison, lapin, train, lait, fleur, poisson	2	21.5%	V0 + faire
V2	32	18.7%	V1 + trou, oiseau, lit, cheval, gâteau, oreille, chat, éléphant, jeu, place, bouche, chien, morceau, chambre, pomme, doigt	3	26.6%	V1 + garder
V3	64	28.1%	V2 + poussin, canard, poule, carte, verre, montre, matin, monsieur, yeux, vache, boîte, camion, porte, oeuf, biberon, sac, rose, caméra, page, chausson, image, ballon, animal, assiette, mouchoir, cuillère, chanson, bras, fille, table, feuille, banane	6	34.6%	V2 + mettre, dire, tenir
V4	128	39.5%	V3 + mouton, balle, chaussure, bout, souris, bouton, bateau, téléphone, musique, carotte, ferme, nounours, puzzle, enfant, arbre, ours, chaise, mamie, soleil, cheveu, papillon, tour, souffle, tasse, fil, panier, café, bonhomme, chapeau, lettre, lumière, soeur, terre, pelle, dent, cochon, pantalon, vélo, sapin, jouet, fenêtre, école, forme, fruit, avion, garçon, crocodile, miette, argent, crèche, chaussette, château, photo, dessin, ventre, colle, clown, renard, pot, cuisine, lune, tétine, neige, tapis	12	41.9%	V3 + prendre, venir, manger, jouer, appeler, trouver
Vm	2159	100%	All nouns in the corpus	860	100%	All verbs in the corpus

It might be important to note that for our model, we used the classical notation of nouns and verbs, but that we could as well have referred to object-referring-words and action-referring-words, if it weren't for the fact that we used a syntactic POS-tagger to evaluate the model's outcome. In principle, the model could work with other categories, such as finer-grained noun categories (e.g., animate/inanimate, human/non-human, edible/non-edible), or finer-grained verb categories (e.g., causative verbs, etc).

### Training and Testing

We divided the corpus into training and test sets. To evaluate the robustness of the model, we first split the corpus into ten mini-corpora (each of them containing a tenth of the total corpus), then split each of them into a training (two thirds of the mini-corpus) and a test corpus (one third of the mini-corpus). This manipulation that leads to small non-overlapping corpora allows us to compute the variability of the model's performance, over each of the 10 runs.

To train the model, we collected the frequencies of each sequence of bigrams and trigrams of words encountered in the training corpus. In principle, our model relies on trigram frequencies, but in the test phase, when it makes predictions about unknown words, it relies on bigrams if the trigram that forms the context of this word has not been encountered during the training phrase. An example of how the model counts trigrams in an utterance is given in Table 2. Utterance boundaries (transcribed as strong punctuation in the corpus, coded as “*{*” and “*}*”) were used as elements of context, but no n-gram could span over such boundaries (for example in “*Take that. Yes, that*,” the 3-gram “*that } {*” is not counted).

**Table 2:** The trigrams that are counted for the sentence “*Mais regarde, le bébé éléphant il va manger*.” (*But look, the baby elephant is going to eat*.).

**Framing context**		**Left context**		**Right context**	
**Context**	**Target Word**	**Context**	**Target Word**	**Context**	**Target Word**
{ _ regarde	mais	{{ _	mais	_ regarde }	mais
mais _ }	regarde	{ mais _	regarde	_ }}	regarde
{ _ bébé	le	{{ _	le	_ bébé éléphant	le
le _ éléphant	N	{ le _	N	_ éléphant il	N
bébé _ il	éléphant	le bébé _	éléphant	_ il va	éléphant
éléphant _ va	il	bébé éléphant _	il	_ va manger	il
il _ manger	V	éléphant il _	V	_ manger }	V
va _ }	manger	il va _	manger	_ }}	manger

*The words ‘bébé’ and ‘aller’ (meaning respectively ‘baby’ and ‘to go’) are in the semantic seed*.

*Projection Tier: { mais regarde le N éléphant il V manger }*.

*Tokens: { mais regarde le bébé éléphant il va manger }*.

#### Testing

During the test phase, the n-gram frequencies learnt during training, together with the local context of target words, were used to predict their syntactic category. To make a prediction, the context of the target word was compared with the set of n-grams collected during training. If this specific two-word context had been encountered during training as part of at least one trigram, the model selected as its prediction the most frequent item completing the trigram. If no trigram featured this two-word context, the process was reiterated with only one word of context (the left one for framing contexts). In a case where the one-word context was never encountered as part of a bigram, the model did not attempt to make a prediction.

One may note that our choice of model is extremely simple, since it consists of a table of trigrams, and does not attempt to assign probabilities to unseen events, as do more sophisticated models typically used in Natural Language Processing (e.g., deep-learning models, Markov chains, or regression models). The main reason for this choice is the interpretability of the model's parameters. The chosen framework allows us to easily analyze which contexts do most of the job (to glimpse ahead: those with pronouns for verbs and those with determiners for nouns). This would not have been the case using other models, for instance, neural networks (besides, the corpora we used are probably too small to train a neural-network). The simplicity of the model also makes the comparison between left, right and framing contexts extremely easy. A final argument in favor of our algorithm is that despite its simplicity, it is very effective. This suggests that infants do not need highly complex calculations to use statistical information from contexts.

#### Targets

To test the model, we took an unseen part of the corpus. As was said earlier, the model did not attempt to make a prediction for each word in the corpus. Rather, target words for which the model attempted a prediction had to fulfill the following two conditions: first, the context word closest to the target must have been seen by the model during training. In other words, the model did not attempt a prediction when it had no information on which to base its prediction. Second, target words should not be too frequent. In practice, words that had a frequency of 0.05% or more during training were excluded from categorization (corresponding to having been encountered 17 times or more during training). At this threshold, most function words were excluded, while most content words remained suitable candidates for categorization (more precisely 97.53% of the noun types and 94.63% of the verb types were selected, and among the few excluded nouns and verbs, most belonged to the smallest semantic seeds and were consequently known by the model).

### Evaluation

To evaluate the model's performance, we calculated precision and recall for the noun and verb targets (see below) and compared the performance of the context-aware models (left, framing and right) to a chance model that constitutes a baseline for our experiments.

#### Precision and Recall

The use of the semantic seed entails that the training corpora contain some categorized words (N or V, the known words from the semantic seed), and a lot of tokens for which the category remains unknown (articles, adjectives, adverbs and the vast majority of the nouns and verbs that are not in the semantic seed). This fact has a consequence on the set of possible responses the model can produce in the categorization task. Because the model chooses as its response the most frequent item that was encountered in a given context, it may respond either with a category (N, V), or with a specific word-form (see Table 3 for an example).

**Table 3:** Example of how the left context model would decide how to categorize a target word in two different scenarios.

Semantic Seed		
N: baby, blankie, bottle		
V: go, do		
Context: *{ the _*		
	Trigram counts from training	
	**Scenario 1**	**Scenario 2**
‘*{ the giraffe*’	2	4
‘*{ the baby*’	4	2
Model's Prediction	N	giraffe

*If the model encountered the following trigrams during training: “{ the giraffe” twice and “{ the baby” 4 times, with ‘baby’ in the semantic seed, then the left context “{ the _” will trigger the prediction “N”, since it is the item encountered most frequently within this context. If, in contrast, “{ the giraffe” had been encountered more frequently than “{ the baby”, the model would have predicted “giraffe” to occur in the context of “{ the _”*.

In this way, the model's responses were coded into three categories: noun, verb, and other. They were compared to the actual category present in the test corpus and used to compute hit, miss, and false alarm rates, separately for nouns and verbs. A hit was recorded whenever the model's response was either “*N”* or “*V”* and matched the actual category of the target word. A miss was recorded when the model should have responded “*N”* or “*V”* but instead replied something else, for example “*giraffe”* or “*V”* when the correct answer was “*N.”* A false alarm (FA) was counted when the model responded “*N”* or “*V*,” whereas the target did not belong to that category. We should note that wrongly responding “*giraffe”* leads only to a miss (for nouns) but answering “*N”* when the correct answer is “*V”* leads to a miss for verbs and a false alarm for nouns.

These measures enable us to compute the precision and recall of the model. Precision is the hit rate divided by the total number of responses of a given category: hit/(hit + FA). If the precision is high, this means that when the model responds noun (or verb), it is usually correct. Recall is the hit rate divided by the total number of target words from a given category in the corpus: hit/(hit + miss). A high recall means that most of the nouns (resp. verbs) present among the target words have been categorized as such by the model.

#### Baseline: Chance Model

**Figure 3:**
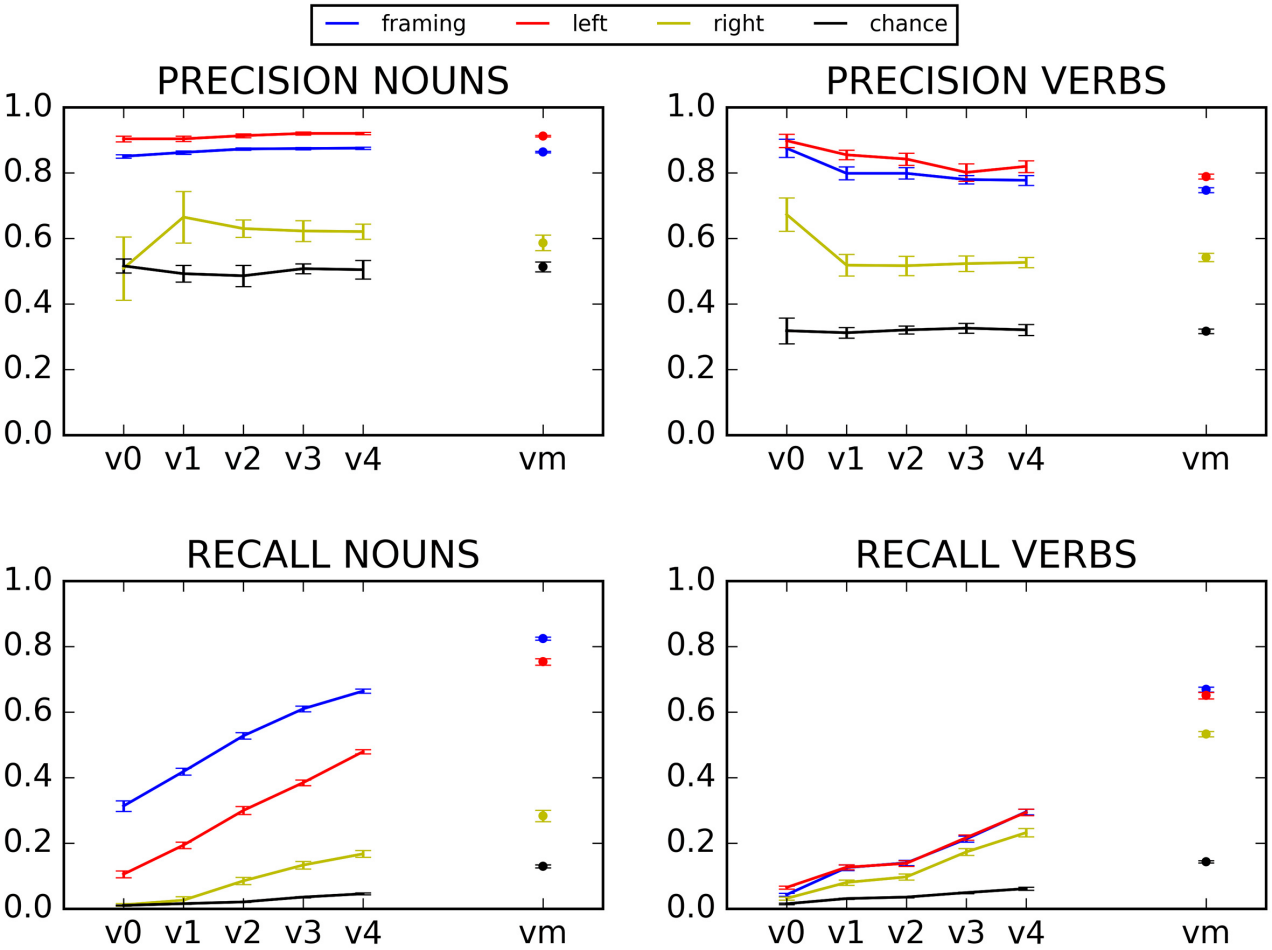
Precision and recall for N and V, for various semantic seed sizes. Error bars represent the standard error of the mean of the ten different mini-corpora.

To evaluate objectively the performance of the learning mechanism, we created a different model that plays the role of a baseline. This model randomly categorized nouns and verbs without taking into account the context of the target words. The only information available to this model was the number of projection of nouns and verbs in the training corpus, which varies according to the size of the semantic seed. For example, if the training corpus contains 10% of known verbs, 10% of known nouns and 80% of words belonging to other categories, the baseline model randomly attributes a verb category 10% of the time, a noun category 10%, and neither noun nor verb for the remaining 80% of the words. For this model—as for the others—we computed the precision and the recall for the noun and verb categories, and we did this 10 times, using the 10 mini-corpora. Note that contrary to the other three models which are deterministic, the baseline model contains a chance component, which means that running the model twice over the same corpus will yield slightly different results. It turns out that the performance of the chance model is stable over the 10 mini-corpora (see Figure 3), so that we estimated that running the baseline model several times over each mini-corpus was not necessary. If the two-word local contexts contain useful information for noun/verb categorization, then the context-aware models should exhibit a better performance than the chance model.

## Results

We first present here the results for the main categorization task, the precision and recall for nouns and verbs, for various semantic seed sizes and the four models we implemented (left, framing, right and chance). Then, we present some *post-hoc* analyses conducted to better understand the behavior of the models: an analysis of the misses for the smallest semantic seed, and a table presenting the most frequently used contexts.

### Precision and Recall

The precision (top) and recall (bottom) of the left context (red), right context (yellow), framing context (blue) and chance (black) model are presented in Figure 3, with nouns on the left side and verbs on the right side. The x-axis in all graphs represents the different semantics seeds.

We ran mixed effects models in R (R Core Team, 2013) with the package lme4 (Bates and Sarkar, 2007; Bates et al., 2015). The statistical models we created aim to analyze the relation between our measures, precision and recall (*precision* [0–1], *recall* [0–1]*)* and the predictor variables: model type (*model:* baseline, right, left, framing), semantic seed size, (*voc*: V0, V1, V2, V3, V4, Vm), and the targets: nouns or verbs (*n_v*). Random intercepts and slopes for the 10 mini-corpora (the *fold)* were modeled for the predictor variables semantic seed size (*voc*) and noun or verb targets (*n_v*). This resulted in the following model:

precision ~ model ^*^ n_v ^*^ voc + (n_v ^*^ voc|fold)

We built a similar model for recall (*recall* [0-1]):

recall ~ model ^*^ n_v ^*^ voc + (n_v ^*^ voc | fold)

In order to be able to compare all types of models against each other, we repeated our analyses three times, changing every time the base value of the *model* variable (either right, left or framing). This resulted in a total of six mixed models, accounting for the 2 measures, and therefore we adapted our level of significance to 0.05/6 = 0.0083 instead of 0.05, according to a Bonferroni correction. Visual inspection of residual plots did not reveal any obvious deviations from homoscedasticity or normality. The full output of all models can be found in the Supplementary Material of this article (Data Sheet 1).

Overall, the left context and framing context models typically yield better precision than the baseline (precision: left model: β = −3.675e−01, *t* = −9.804, *p* < 0.001, framing model: β = −3.233e−01, *t* = −8.626, *p* < 0.001). The right context model performs more poorly, with no significant overall difference in precision relative to baseline (β = −0.012865, *t* = −0.343, *p* = 0.73).

The first striking result is the excellent precision that is obtained by the left and framing models, independently of the size of the semantic seed, which was not a significant predictor variable when modeling the precision of the left and the framing context models (β = 2.899e−03, *t* = 0.368, *p* = 0.71, and β = 2.455e−03, *t* = 0.312, *p* = 0.76, respectively). Precision is above 80%, for nouns and verbs, for both models. This means that even when the semantic seed is very small, and only a small number of contexts can be learned, these contexts are good contexts, that provide error-free categorization. In contrast, recall depends highly on the number of nouns and verbs categorized in the training corpus, with a low recall when the semantic seed is small, and a clear improvement as it increases (β = 0.118103, *t* = 24.435, *p* < 0.001)^2^. This reflects the fact that with a small semantic seed, the model can learn only a limited number of noun and verb contexts, and consequently, that it can categorize only a limited number of new nouns and verbs (albeit with a good precision).

The kind of contexts used by the model impacts the results. The right-context model is clearly the least efficient at correctly predicting nouns and verbs, with both precision and recall significantly lower than the other two models (β = −3.546e−01, *t* = −9.461, *p* < 0.001; β = −3.104e−01, *t* = −8.283, *p* < 0.001, for the right model compared to the left model and the framing model respectively). The others two models, relying on left and framing contexts, exhibit consistently good results, with a precision far above the baseline at all semantic seed sizes, as indicated above (~0.9 for nouns and ~0.8 for verbs), and a recall that rapidly rises above baseline as the semantic seed grows (results for the interaction of semantic seed size and model type when comparing the baseline model and the left model: β = −0.097120, *t* = −14.208, *p* < 0.001; results for the interaction of semantic seed size and model type when comparing the baseline model and the framing model: β = −0.074068, *t* = −10.836, *p* < 0.001). The performance of these two models is very similar, with a small, nonsignificant advantage for the left model for noun and verb precision (β = 4.416e−02, *t* = 1.178, *p* = 0.24), and a rather large significant advantage of the framing model for the recall of nouns (β = 0.028534, *t* = 2.952, *p* < 0.004).

Finally, the framing and left context models exhibit a better precision for nouns than for verbs (although this does not reach significance, β = −0.098109, *t* = −1.851, *p* = 0.05 when we look at the interaction between the left model and the verb category and β = −0.096205, *t* = −1.815, *p* = 0.07 when we look at the interaction between the framing model and the verb category), and recall is also higher for nouns (significant difference when taking the framing model as a base level: β = 0.356097, *t* = 13.377, *p* < 0.001). This difference between nouns and verbs might come from the fact that the syntactic dependents of a noun are generally closer to their head than is the case for verbs [a similar advantage for nouns over verbs was observed in Bannard et al. (2009), in a model of young children's productions]. This is also consistent with the developmental literature, since nouns are typically understood and produced earlier than verbs (Gleitman, 1990; Waxman and Markov, 1995; Gentner, 2006; Bergelson and Swingley, 2012, 2013). It should also be noted that precision varies slightly more for verbs than for nouns (larger error bars for verbs for the framing and left context models), this is probably due to the lower recall for verbs (lower recall is caused by less hits and variance increases for lower numbers). Furthermore, the category of verbs is more heterogenous than the one of nouns: typically, we can describe a verb as intransitive, transitive, ditransitive, modal, stative, dynamic, etc. The syntactic selection of these different types of verbs influences the context they appear in. The variety inside the class of verbs and the low number of verbs in the smallest sizes of the semantic seed can also explain why the precision of verbs decreases a bit with the growth of the semantic seed throughout our experiences (although not significantly, as stated above). Because the smaller semantic seeds are only composed of 1, 2 or 3 verbs, these verbs might lead to more homogenous contexts than when more verbs are added.

### Error Analysis of Misses

Since the recall was low for the smallest semantic seed, there were many misses: this is the reason why we focused our analysis of the model errors on the misses. The very high precision, on the other hand, means that false alarms were very rare. Our study of misses allows us to investigate what our model predicts when it should predict “*N”* or “*V”* and fails to do so.

**Figure 4:**
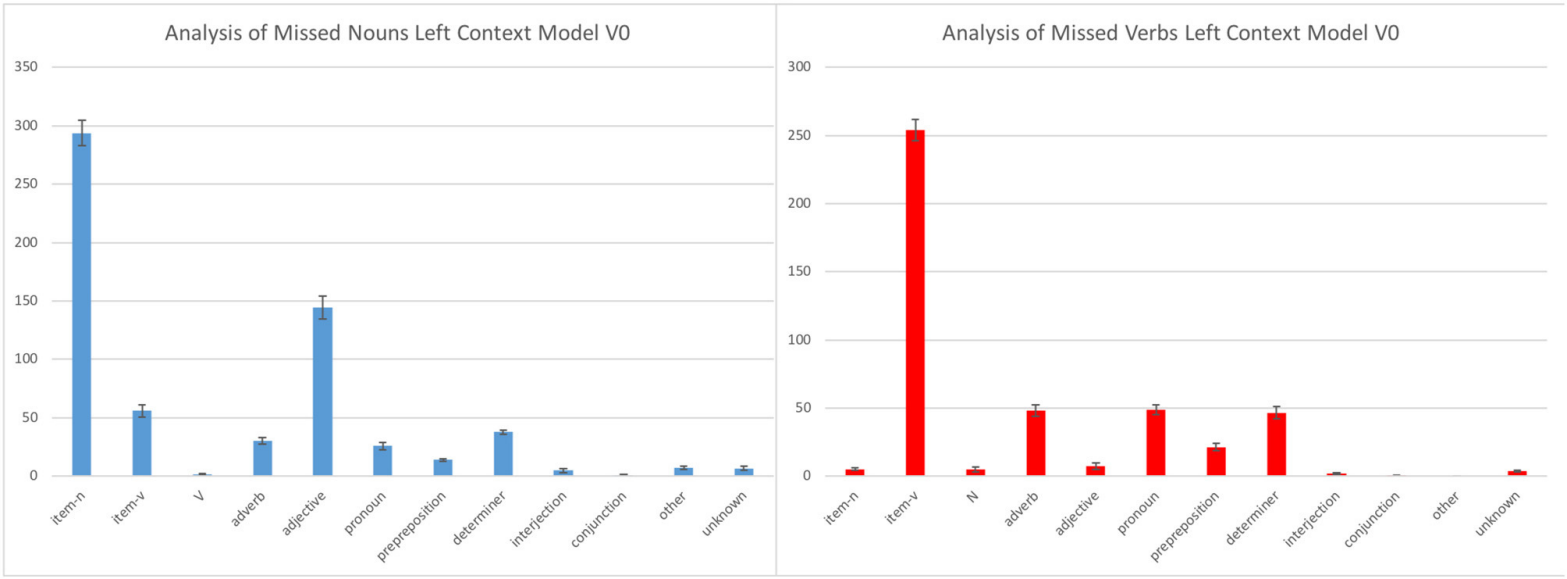
Misses of the left context model with the smallest vocabulary size.

Figure 4 presents the misses of the left model with the smallest vocabulary size (V0)^3^. The graphic on the left represents the noun misses (cases where the test corpus contained a noun, and something else than “*N”* was predicted). In Figure 4, we group together the different responses given instead of “*N.”* Since the model could give as response either “*N*,” “*V*,” or a specific wordform (e.g., *giraffe, slowly, carry, not…)*, we classified the errors that involved specific wordforms using classical categories: *item*-N and *item*-V for specific nouns and verbs (to distinguish them from the N and V categories built around the semantic seed), and adjective, adverb, pronoun, preposition, etc. for all other specific wordforms. The graphic on the right gives the corresponding results for verbs misses.

The most common type of miss is the prediction of a specific item of the correct category (“item-N” for nouns and “item-V” for verbs), which means that the model confuses specific items with their actual category. Developmentally, this type of error has the least negative impact for an infant. As can be expected, the number of such errors decreases with the number of verbs and nouns in the semantic seed^4^ (congruent with the fact that the recall increases with the size of the vocabulary).

The other types of misses are much less frequent. When the model misses a noun and does not predict a specific noun item, its answer is most of the time an item of the adjective category. This is perfectly plausible, as a lot of frequently used adjectives in French are placed in between determiners and nouns. For example, when we have a context such as “*voit le _”* (“*sees the _”*), the word in the gap could perfectly be an adjective as well as a noun: “*voit le petit lapin”* (“*sees the little rabbit”*). The misses that are caused by “*item-V”* can also be explained by some specific contexts, such as the “*veut le _”* context (“*wants the”* or “*wants to _ him”*): it can be followed by a verb, as for instance in “*Marie veut le caresser* (‘*Mary wants to pet him’*), or by a noun, as in ‘*Marie veut le poney*’ (‘*Mary wants the poney”*).

When a miss is recorded for a verb and the model does not predict a specific verb its answer is most of the time an adverb, a pronoun or a determiner. As for the misses for nouns, these guesses can be explained by contexts that can also receive these categories, such as “*Marie veut _”* (“*Mary wants _”*). It can be completed by either a verb, an adverb, a determiner, or a pronoun: “*Marie veut* *****danser*****” (“*Mary wants* *****to***** *****dance*****”), “*Marie veut* *****bien***** *danser”* (“*Mary would* *****gladly***** *dance”*), “*Marie veut* *****un***** *poney”* (“*Mary wants* *****a***** *poney”*), “*Marie veut* *****le***** *caresser”* (“*Mary wants to pet* *****him”*****).

### Frequently Used Contexts

In this subsection, we examined the contexts most frequently used by the left-context model to classify noun and verb targets. The qualitative study of these contexts helped us understand why the model performs well and what its pitfalls are.

The contexts are represented in Table 4. In each subtable, the first column gives the most frequently used contexts (ordered by decreasing frequency), the second one the translation, the third and fourth ones the number of times the model used this specific context during the test (2 columns giving the number of times this context was followed by a noun or by a verb) and finally the answer chosen by the model whenever it encountered this context. Thus, an “*N”* in the last column of the first table, along with a large number in the fourth column is evidence that the model gives a correct answer most of the time. For example, for the “*{ un _”* (“*{ a _”*) context, which is the most frequent context used by the model when categorizing nouns, out of the 179 encounters of this context, it was followed by a noun 170 times in the test corpus, and only once by a verb (the remaining times it was followed by something else, adjectives or adverbs). Since the model predicted “*N”* whenever it encountered this context, this means that it gave a correct answer 170 times, and a false alarm for the noun category 9 times. The same reasoning applies to the verb contexts.

**Table 4:** Most frequent contexts used by the left context model during categorization, with a maximal projection (Vm).

**Context**	**Translation**	**Number of Uses**	**Target = N**	**Target = V**	**Answer from Model**
**Most Frequently Used Contexts Used to Predict Noun Targets**					
{ un	{ a	179	170	1	N
est un	is a	144	135	1	N
{ le	{ the	133	124	3	N
{ une	{ a	121	107	3	N
dans la	in the	105	101	3	N
de la	from the	109	101	5	N
{ les	{ the	103	97	2	N
{ la	{ the	93	88	2	N
est le	is the	92	83	0	N
dans le	in the	79	79	0	N
est une	is a	89	79	4	N
un petit	a little	79	78	0	N
à la	to the	78	75	0	N
sur le	on the	67	64	0	N
{ des	{ some	62	59	0	N
sur la	on the	56	54	0	N
est la	is the	66	49	2	N
à l'	to the	49	46	3	N
le petit	the little	44	44	0	N
c' est	it is	333	43	42	pas (*not*)
**Most Frequently Used Contexts Used to Predict Verb Targets**					
{ tu	{ you	323	41	258	V
{ on	{ we	133	12	110	V
tu as	you have	143	36	93	V
on va	we are going to	87	0	75	V
{ il	{ he	83	7	67	V
il est	he is	121	7	67	pas (*not*)
{ ça	{ it	86	8	62	V
que tu	that you	75	11	52	V
tu veux	you want	55	1	50	V
{ je	{ I	50	0	44	V
tu me	you me_(directobject)_	51	6	43	V
c' est	it is	333	43	42	pas (*not*)
qu' on	that we	59	2	42	V
tu le	you it_(directobject)_	52	12	40	V
tu te	you yourself_(directobject)_	47	6	40	V
tu vas	you are going to	49	0	40	V
je te	I you_(directobject)_	41	2	36	V
tu t'	you yourself_(directobject)_	33	0	33	V
on le	we it_(directobject)_	40	9	29	V
{ elle	{ she	36	1	29	est (*is*)

We can note that the 20 most frequently used contexts for “*N”* all include at least one function word; more specifically the 19 most frequently used contexts contain a determiner. This is potentially not surprising given the crucial role played by function words in grammatical structure; yet no concept of function word was built in our model, let alone a concept of determiner. This means that the sheer frequency of function words, together with their distributional properties, were sufficient to make function words a key ingredient for the efficient discovery of the noun category. We find a similar situation for verbs, where this time the most useful cues are pronouns, which occur in 20 contexts out of 20.

It is interesting to note that the most frequent contexts for verb targets also feature some contexts predicting the negation particle “*pas.”* Indeed, in French, this small word is often considered as belonging to the category of adverbs, but is placed in the same position as a verb when we only consider the two-word context to the left, especially since in natural speech the pre-verb particle “ne” is often dropped (“Je veux pas” *I don't want* vs. “Je veux manger” *I want to eat*). The fact that the left context model predicts “*pas”* for some very frequent contexts during the maximal vocabulary (Vm) experiment, explains (partly) why a hundred percent recall is not reached even in this condition.

Furthermore, these contexts show why the precision for nouns is higher than for verbs. When we look at the number of verb targets among the contexts that are used most frequently to predict nouns, we globally observe a lower number than when we look at the noun targets among the contexts that are used most frequently for the prediction of verbs. Indeed, most of the time, a context such as “*tu as _”* is followed by a verb. However, about a third of the “*tu as _”* contexts are followed by a noun (for example in “*tu as faim”;* literally, “*you have hunger*,” meaning “*you are hungry”*). Nevertheless, the model classifies all targets in this context as a verb, leading to 36 false alarms in this case and thus to a lower precision for verbs than for nouns.

## Discussion

We presented a learning mechanism aiming to explain the formidable ability of infants to guess the probable meaning of unknown words by using their syntactic contexts. To do this we implemented a computational model that aims at categorizing nouns and verbs on the basis of their local contexts. Our algorithm is driven by frequency and expectation. We compared three different types of contexts and showed that both left and framing contexts were effective, whereas the right context gave poor information to predict categories. Overall, this model demonstrates that relying on local contexts and on a semantic seed is an efficient and simple method that may allow children to learn which contexts correspond to nouns, and which to verbs, as demonstrated with infants in several psycholinguistic experiments (Cauvet et al., 2014; Shi, 2014; Brusini et al., 2017; Babineau et al., 2020).

This model rests on two assumptions, that we argue are highly plausible. First, infants are supposed to be able to build a semantic seed. The semantic seed is a handful of words for which infants have succeeded in learning a meaning (frequent words, referring to concrete objects and actions, presented in pragmatically helpful situations), and that they are able to group together: a small number of known object-referents to form a proto-category of nouns and a few known action-referents to form a proto-category of verbs (Carey, 2009). Second, the model rests on the assumption that infants keep track of bi- and tri-gram frequencies, a hypothesis supported by many experiments (e.g., Santelmann and Jusczyk, 1998; Gomez and Gerken, 1999; Höhle et al., 2006; van Heugten and Johnson, 2010; Marchetto and Bonatti, 2013). The number of nouns and verbs supposedly known is very low: only 8 nouns and 1 verb at the smallest size of the semantic seed, a vocabulary which might plausibly be known by infants around the age of 10–12 months. Bergelson and Swingley (2012, 2013) present data suggesting that 10–13-month-olds already know 2 verbs, while 9-month-olds already know 10 nouns, rendering our initialization hypothesis highly plausible. We showed here that as soon as infants are able to group known words on semantic grounds, the use of local contexts is highly efficient to spread these proto-categories to many unknown words. We suspect that such a mechanism would be just as efficient on the learning of syntactic categories other than nouns or verbs: whenever there is a link between a semantic feature and a local morphosyntactic context, young children could rely on the local contexts to spread this semantic feature to other, yet unknown, words. Consistent with this hypothesis, a large-scale cross-linguistic study of the kind of semantic features that are commonly encoded in morphosyntax revealed that these correspond to core knowledge distinctions, that are perceived very early by infants (e.g., the mass/count distinction, or animate/inanimate, Strickland, 2017). Our experiments demonstrate the interest of computational approaches in developmental and cognitive science, as the models we built allowed us to evaluate different cognitive mechanisms in an efficient manner and confront their outcomes with results from experimental work. The model possesses two important characteristics that make it particularly attractive as a model of early lexical acquisition: the efficiency of the semantic seed, and the fact that it categorizes words in context. As we saw above, the semantic seed is highly plausible, and it is also highly efficient: even at the smallest size of the semantic seed, the model already achieves an excellent precision, both for nouns and for verbs. Unsupervised learning algorithms seeded with semantic information have been presented before in the computational linguistic literature (to solve other problems), with excellent results (Yarowsky, 1995). Arguably, we can oppose that the method presented here is not a complete mechanism for bootstrapping the nouns and verbs categories. Indeed, the models we presented here do not use the words they managed to categorize in order to expand their semantic seed to learn even more categorizing contexts, something we would expect real learners to be able to achieve.

The second important characteristic of the model is that it categorizes words in context. It does not attempt to build a “mental dictionary,” a list of word-forms, where each word-form would be assigned a syntactic category—or several possible ones for each possible meaning. Instead, the model categorizes words solely on the basis of their immediate context (whenever it is sufficiently informative). This feature buys the model two important advantages: first, novel words, that are encountered for the first time, can be categorized (provided they occur in a known context). This is important as it means that a child could deduce the category of a word she/he heard for the first time and use it to guess the meaning of the novel word, as has been observed in many infant experiments (Bernal et al., 2007; Waxman et al., 2009; Oshima-Takane et al., 2011; He and Lidz, 2017; de Carvalho et al., 2019). Second, the model does not suffer from the fact that many words possess more than one syntactic category, in fact, it does not even notice such cases. This particular aspect of the model's behavior is also consistent with recent experimental work testing how toddlers handle homophones: not only do 20-month-olds understand noun-verb homophones in their native language (Veneziano and Parisse, 2011; de Carvalho et al., 2017), they are also willing to learn a novel meaning for a word-form they already know (e.g., “to give”), provided that the novel word appears in a context that would be inappropriate for the known meaning, e.g., it belongs to a different syntactic category (e.g., they can taught that *a give* is the name of a novel animal; Dautriche et al., 2015, 2018).

These two characteristics, that mesh well with the developmental literature on word learning in infants, gives a real plausibility boost in favor of the present model, compared to previous work relying on local contexts for categorization, at least at the earliest stages of learning. For example, the Redington et al.'s model yielded fine-grained syntactic categories (much more precise than simply noun vs. verb), but attempted categorization only on the most frequent words of the corpus, the words that a child would have heard many times in her input. As a result, this model would not even have attempted to categorize a word on first encounter. Since it builds a diagram of similarities between word-forms, it also ignores word homophony and falls back on assigning to each word-form the syntactic category that is most frequent, at the risk of confusion (e.g., a ring, to ring). One might think that the two approaches could be usefully combined by children: on one hand, an on-line categorization approach based on immediate context, as in the present model, could provide infants with a first hint as to the possible meaning of a word (even on first encounter); on the other hand, the fine-grained categorization provided by the analysis of a large number of contexts (as implemented in Redington et al., 1998) could give slightly older children more precise information about a word's meaning, which could be especially helpful for acquiring the meaning of verbs (Gleitman, 1990; Naigles, 1990; Yuan and Fisher, 2009; Arunachalam and Waxman, 2010), or of some other more abstract words (e.g., quantifiers, preposition, etc., see Waxman and Lidz, 2006).

The present model also improves over the Frequent Frames model proposed by Mintz (2003), from which it was partly inspired. The Frequent Frames model also aligns with developmental data and has the capacity to categorize a word on first encounter, provided the context is known (indeed, this characteristic was borrowed from the Frequent Frames model). Its main drawback is the fact that it builds several classes for each syntactic category: for instance, the frames “*the _ is”* and “*a _ is”* both select nouns. The present model escapes this difficulty through seeding the categorization process with a few known words, which are categorized precisely because we supposed their meaning known (objects and actions). Not surprisingly, adding more information in the input yields a better performance in the end.

The *post-hoc* analysis of the most frequently used contexts demonstrated that the efficiency of the model is in a great part due to function words. These words play an important linguistic role in the structure of sentences. Many experiments have demonstrated that infants notice these words early in development, thanks to their acoustic and distributional characteristics (Shady, 1996; Shafer et al., 1998; Shi et al., 1998, 2006a,b; Shi and Lepage, 2008). Then, from around 14–18 months of age, infants can use them to build expectations about novel words (Bernal et al., 2007; Shi and Melançon, 2010; Brusini et al., 2016; Babineau et al., 2020). Here, the algorithm used by the model did not attribute any specific role to these words, but their frequency and their natural pertinence regarding the categorization task enhanced their role naturally. This alignment between what we know of toddlers processing of function words, and the way they are used by our model, confirms its developmental plausibility regarding the acquisition of the noun and verb categories. Additionally, the results presented here also show that it is not necessary to form categories of function words, such as determiner or pronoun, to be able to use them to predict nouns and verbs. The idea that children group function words together into categories is rather intuitive (Shi and Melançon, 2010) but remains disputed (Pine and Martindale, 1996; Valian et al., 2009; Pine et al., 2013; Yang, 2013). Here, we demonstrated that this step is in fact unnecessary. The simple knowledge of the phonological form of the function words could be enough to bootstrap the growth of content word categories. Here, we see how the use of modeling work enlightens current developmental hypotheses.

For our research, we compared three types of context: left, right and framing. We found that the left context leads to the best precision. Two hypotheses might be proposed to explain why. The first is that many of the most frequently-used contexts (see Table 4) include a marker of the beginning of the sentence. Indeed, a determiner such as “*le”* or “*la”* (“the”) is homophonous with clitic object pronouns in French (“*him/her”*). Knowing that “*le”* or “*la”* (“the”) is placed at the beginning of the sentence gives crucial information that the function word is a determiner and consequently likely to be followed by a noun (or an adjective). Another explanation for the better performance of left contexts would be that French, like English, is mostly right-branching: there is a large number of syntactic phrases in which the head is at the beginning (right-branching phrases are also called head-initial phrases). Since heads are by definition words that constrain the category of the phrase and the nature of their dependents, it can be expected that finding the head at the left edge of the phrase is very informative, and, accordingly, that in general words located on the right of the target will be much less informative. Since French comprises both left-branching and right-branching structures (albeit skewed in favor of right-branching ones) it might favor both left and framing contexts. If this analysis is correct, we expect that we would get different results for languages in which the distribution of left-branching and right branching structures is different. In this respect, it would be interesting to do the same study with a language such as Japanese, which is well-known to be almost fully left-branching.

Despite all the qualities of the *semantic seed* model, the way it is currently implemented, it possesses several characteristics that lack psychological plausibility: (1) it has a perfect memory; (2) it has no way of increasing its vocabulary of known words; and (3) it works from an input segmented into words. We think that none of these aspects are crucial for the good performance of the model, and that each could be modified to make it more plausible (and perhaps even further improve its performance). We will discuss each of these in turn. First, as currently implemented, the model never forgets any of the word triplets presented during training, thus assuming perfect memory on the part of the infant (which is clearly undesirable). However, since the model's performance relied on those word triplets which had been encountered most frequently, it should be possible to incorporate a forgetting mechanism through which triplets which have been encountered only a few times (in a to-be-defined number of utterances) are forgotten. This would probably not impact the performance too dramatically (as an aside, most models suppose perfect memory to test the feasibility of a method; e.g., Redington et al., 1998).

Second, the model currently has no way to increase its vocabulary. It starts out with a small initially known vocabulary (the semantic seed), memorizes word triplets from the training corpus, then uses these to categorize content words. Ideally, the model should be able to rely on its high precision to learn from its own predictions a new set of newly-learned words, perhaps with a simple threshold of confidence (although we should note that real learners would presumably exploit the categorizing that they performed in order to learn something about the semantics of the words they categorized, before adding them to their semantic seed). In that way, the model could perhaps start out with the smallest semantic seed (which already demonstrates a high precision), and increase the number of words it categorizes, namely the recall, by accumulating new contexts, precisely the ones it can extract thanks to the newly-learnt words. Thus, the model could start with as little as 8 nouns and 1 verb, and categorize many more words in an iterative fashion.

Third, the model takes as input a transcribed corpus (like all other computational models attempting to categorize lexical items so far), and it therefore assumes that the continuous speech stream is segmented into words. This is a reasonably plausible assumption in light of the many experiments showing that infants already possess rather refined word-segmentation abilities within their first 18 months of life (Jusczyk and Aslin, 1995; Gout et al., 2004; Nazzi et al., 2005, 2006; Fló et al., 2019), although we do not know when exactly children might have access to an adult-like segmentation of speech (Ngon et al., 2013). Future work should ideally attempt to start from an unsegmented input and adopt a plausible word-segmentation strategy as a first step (Johnson et al., 2015). Last, a final improvement of the model could be to use grammatical categories with maximal cognitive plausibility. In the present experiments, we chose to work with the noun and verb categories for three reasons. First, the experimental literature reviewed in the introduction shows that 18-month-olds are able to exploit local contexts to map nouns to objects and verbs to actions (e.g., He and Lidz, 2017). Second, and this is a practical reason, nouns and verbs can be identified by off-the-shelf part-of-speech taggers. Third, these categories seem to be generally present cross-linguistically. However, we are well aware that these categories are not necessarily universal (Feng et al., 2020), and definitely not homogeneous. The verb category is an ideal example of that: verbs can be divided in numerous subcategories for which children have some sensitivity, for example 1-participant action verbs vs. 2-participants action verbs (Yuan and Fisher, 2009).

More generally, we think that the mechanism tested in our model would be relevant for any categories, not just nouns and verbs: namely, using known content words to learn about the contexts they appear in, then, whenever a novel content word is encountered, using these contexts to project some of the properties of the known content words on the novel content word. For instance, some languages implement specific morphology for the animate/inanimate distinction, mass/count, human/non-human, and so on. Infants learning these languages could exploit these markers to narrow down their hypotheses about the meaning of words occurring in these contexts. Consistent with this hypothesis, a large-scale cross-linguistic study of the kind of semantic features that are commonly encoded in morphosyntax revealed that these correspond to *core knowledge* distinctions (Spelke, 2000), that are perceived very early by infants (e.g., the mass/count distinction, or animate/inanimate, Strickland, 2017). One possible interpretation for this fact is the idea that languages are shaped by the generations of children who acquire them (e.g., Christiansen and Chater, 2008): indeed, morphosyntactic markers that encode semantic distinctions that are relevant and salient for infants (*core knowledge* distinctions), will both be learned more easily, and make language learning easier for infants, since they will be able to exploit these markers to rapidly guess the possible meaning of novel words. This is consistent with many modeling studies showing that natural languages are shaped by acquisition and processing constraints (e.g., Piantadosi et al., 2011; Dautriche et al., 2017), as well as with models of language emergence (e.g., Kirby et al., 2008; Gong, 2011).

Notwithstanding the implementation limitations that we raised above, the model can already be used to make predictions regarding the acquisition of novel words, and these predictions can be experimentally tested in children: For instance, by using well-known words to teach them novel syntactic contexts in their native language, and seeing whether they would be ready to rely on those newly-learnt contexts to categorize novel content words (into object-referents vs. action-referents, for instance). This is precisely what Babineau et al. (2021) did in a recent experiment, teaching two groups of 3- to 4-year-olds a novel function word “ko,” in French; in half the children, “ko” replaced all determiners, and preceded well-known nouns and adjectives (e.g., *ko rabbit, ko little chicken*), in a video where a speaker was playing with toys and telling a story; the other half of the children watched the same video, in which “ko” replaced all personal pronouns, and preceded verbs and auxiliaries (e.g., *ko plays, ko will jump*). At test, all children were presented with a choice of 2 videos, one exhibiting a novel object, and the other one a novel action, while they heard “Regarde! Ko bamoule!” (*look! Ko bamoule*). The results showed that children who had heard “ko” in the position of personal pronouns looked more at the novel action than children who had heard “ko” in the position of determiners, who looked more at the novel object. These results thus suggest that young children, just like the model, are able to exploit content words they already know, in order to learn some of the properties of novel function words, then use these novel function words to guess the probable meaning of an unknown content word (*bamoule*). Although this experiment was performed with rather “old” children (3–4-year-olds) and should be replicated with younger children, it already is a very encouraging confirmation of the main hypothesis behind the model.

## Conclusion

The computational model presented here clearly shows the relevance of local contexts to categorize nouns and verbs in sentences. Two crucial characteristics of the current model make it particularly relevant to describe lexical acquisition during infancy. The *semantic seed*—minimal information regarding a handful of known words, grouped into object-referents and action-referents—allows it to group words together with very high precision, even for words that are encountered for the first time (provided they occur in known contexts). And the fact that the model categorizes words in context neatly bypasses the potential difficulties posed by homophones—in this case, noun/verb homophones, which are frequent in many languages. It is noteworthy that, just like adult speakers, toddlers seem to be completely impervious to homophones, not even noticing them: our model behaves in just the same way. Importantly, *any* semantic feature that has a realization in language, can be identified by infants and has the potential to be generalized in that way. The present model thus exhibits a plausible mechanism through which toddlers could succeed in learning about the contexts of nouns and verbs in their native language—knowledge which we know they possess from 18 months on—and perhaps, more generally, could be extended to learning the contexts of more fine-grained categories (such as different subclasses of verbs, adjectives, animates etc.).

## Data Availability Statement

Publicly available datasets were analyzed in this study. This data can be found here: https://phonbank.talkbank.org/access/French/Lyon.html.

## Ethics Statement

Written informed consent was obtained from the individual(s), and minor(s)' legal guardian/next of kin, for the publication of any potentially identifiable images or data included in this article.

## Author Contributions

PB elaborated the idea for this model and conducted a first series of experiments with it for her PhD-thesis at the Laboratoire de Sciences Cognitives et Psycholinguistique under the supervision of AC and PA. OS conducted a second series of experiments for her Master thesis at Université Paris Diderot under the supervision of AC and PA. She recoded the experiments and enhanced Brusini's model. All four authors contributed to the redaction of the manuscript. The figures, tables, statistic analyses, and computer code were elaborated by OS. All authors contributed to the article and approved the submitted version.

## Conflict of Interest

The authors declare that the research was conducted in the absence of any commercial or financial relationships that could be construed as a potential conflict of interest. The reviewer SW declared a past collaboration with one of the authors AC to the handling editor.

## Publisher's Note

All claims expressed in this article are solely those of the authors and do not necessarily represent those of their affiliated organizations, or those of the publisher, the editors and the reviewers. Any product that may be evaluated in this article, or claim that may be made by its manufacturer, is not guaranteed or endorsed by the publisher.

## Footnotes

^1^In pilot experiments, we tested other configurations for the size of the semantic seed, for instance relying solely on frequency for the choice of the semantic seed or implementing a stronger filter to retain only concrete and observable words. The results are highly comparable, the model seems to be very robust with respect to these parameters.

^2^We used the statistical model that uses the left model as the base level for the variable “model,” but the two other statistical models for recall yield similar results at the same level of significance. Please see the Supplementary Material for more details.

^3^We chose the left model because it shows the best performance in terms of precision. We chose the V0 vocabulary because the number of misses is the highest as small semantic seeds lead to the lowest recall.

^4^The data for the other semantic seeds can be found in the GitHub repository.
