# Supplementary material for: The Acquisition of Noun and Verb Categories by Bootstrapping From a Few Known Words: A Computational Model
Source: Front Psychol. 2021 Aug 19;12:661479. doi: 10.3389/fpsyg.2021.661479 (PMC8416756; doi:10.3389/fpsyg.2021.661479)
Supplement: Supplementary file 1 [file Data_Sheet_1.docx]

# Supplementary Materials: Statistical Models

**Statistical model for precision with the left model as base level, output from R:**

summary(model_random_intercept_prec, correlation=TRUE)

Linear mixed model fit by REML

t-tests use Satterthwaite approximations to degrees of freedom ['lmerMod']

Formula: prec ~ model * n_v * voc + (n_v * voc | fold)

Data: scores_models

REML criterion at convergence: -820.3

Scaled residuals:

Min 1Q Median 3Q Max

-6.2026 -0.3867 0.0515 0.3777 5.1599

Random effects:

Groups Name Variance Std.Dev. Corr

fold (Intercept) 0.0058315 0.07636

n_vv 0.0053217 0.07295 -0.89

voc 0.0001568 0.01252 -0.99 0.81

n_vv:voc 0.0001020 0.01010 0.96 -0.98 -0.92

Residual 0.0081050 0.09003

Number of obs: 480, groups: fold, 10

Fixed effects:

Estimate Std. Error df t value Pr(>|t|)

(Intercept) 9.029e-01 3.586e-02 2.600e+01 25.181 < 2e-16 ***

model1 -3.675e-01 3.748e-02 4.460e+02 -9.804 < 2e-16 ***

model2 -3.546e-01 3.748e-02 4.460e+02 -9.461 < 2e-16 ***

model4 -4.416e-02 3.748e-02 4.460e+02 -1.178 0.2393

n_vv 2.559e-03 4.401e-02 4.540e+01 0.058 0.9539

voc 2.899e-03 7.873e-03 5.080e+01 0.368 0.7142

model1:n_vv -2.184e-01 5.301e-02 4.460e+02 -4.121 4.51e-05 ***

model2:n_vv 9.811e-02 5.301e-02 4.460e+02 1.851 0.0648 .

model4:n_vv 1.904e-03 5.301e-02 4.460e+02 0.036 0.9714

model1:voc -9.706e-03 9.624e-03 4.460e+02 -1.008 0.3138

model2:voc 1.039e-02 9.624e-03 4.460e+02 1.079 0.2811

model4:voc -4.445e-04 9.624e-03 4.460e+02 -0.046 0.9632

n_vv:voc -2.298e-02 1.014e-02 1.196e+02 -2.266 0.0252 *

model1:n_vv:voc 2.922e-02 1.361e-02 4.460e+02 2.147 0.0323 *

model2:n_vv:voc -1.537e-02 1.361e-02 4.460e+02 -1.129 0.2595

model4:n_vv:voc 1.317e-03 1.361e-02 4.460e+02 0.097 0.9230

---

Signif. codes: 0 '***' 0.001 '**' 0.01 '*' 0.05 '.' 0.1 ' ' 1

Correlation matrix not shown by default, as p = 16 > 12.

Use print(x, correlation=TRUE) or

vcov(x) if you need it

**Statistical model for recall with the left model as base level, output from R:**

summary(model_random_intercept_rec, correlation=TRUE)

Linear mixed model fit by REML

t-tests use Satterthwaite approximations to degrees of freedom ['lmerMod']

Formula: rec ~ model * n_v * voc + (n_v * voc | fold)

Data: scores_models

REML criterion at convergence: -1161

Scaled residuals:

Min 1Q Median 3Q Max

-2.63530 -0.58922 -0.03702 0.55725 2.96797

Random effects:

Groups Name Variance Std.Dev. Corr

fold (Intercept) 4.958e-15 7.041e-08

n_vv 3.546e-14 1.883e-07 -1.00

voc 2.469e-16 1.571e-08 -0.99 0.99

n_vv:voc 2.361e-15 4.859e-08 1.00 -1.00 -1.00

Residual 4.088e-03 6.394e-02

Number of obs: 480, groups: fold, 10

Fixed effects:

Estimate Std. Error df t value Pr(>|t|)

(Intercept) -0.036601 0.018824 464.000000 -1.944 0.052448 .

model1 0.006285 0.026621 464.000000 0.236 0.813475

model2 -0.026540 0.026621 464.000000 -0.997 0.319291

model4 0.270184 0.026621 464.000000 10.149 < 2e-16 ***

n_vv -0.059976 0.026621 464.000000 -2.253 0.024725 *

voc 0.118103 0.004833 464.000000 24.435 < 2e-16 ***

model1:n_vv 0.071825 0.037647 464.000000 1.908 0.057026 .

model2:n_vv 0.009013 0.037647 464.000000 0.239 0.810892

model4:n_vv -0.296120 0.037647 464.000000 -7.866 2.60e-14 ***

model1:voc -0.097120 0.006836 464.000000 -14.208 < 2e-16 ***

model2:voc -0.066229 0.006836 464.000000 -9.689 < 2e-16 ***

model4:voc -0.023051 0.006836 464.000000 -3.372 0.000808 ***

n_vv:voc -0.018101 0.006836 464.000000 -2.648 0.008369 **

model1:n_vv:voc 0.018638 0.009667 464.000000 1.928 0.054462 .

model2:n_vv:voc 0.053777 0.009667 464.000000 5.563 4.49e-08 ***

model4:n_vv:voc 0.028534 0.009667 464.000000 2.952 0.003320 **

---

Signif. codes: 0 '***' 0.001 '**' 0.01 '*' 0.05 '.' 0.1 ' ' 1

Correlation matrix not shown by default, as p = 16 > 12.

Use print(x, correlation=TRUE) or

vcov(x) if you need it

**Statistical model for precision with the right model as base level, output from R:**

summary(model_random_intercept_prec, correlation=TRUE)

Linear mixed model fit by REML

t-tests use Satterthwaite approximations to degrees of freedom ['lmerMod']

Formula: prec ~ model * n_v * voc + (n_v * voc | fold)

Data: scores_models

REML criterion at convergence: -820.3

Scaled residuals:

Min 1Q Median 3Q Max

-6.2026 -0.3867 0.0515 0.3777 5.1599

Random effects:

Groups Name Variance Std.Dev. Corr

fold (Intercept) 0.0058315 0.07636

n_vv 0.0053217 0.07295 -0.89

voc 0.0001568 0.01252 -0.99 0.81

n_vv:voc 0.0001020 0.01010 0.96 -0.98 -0.92

Residual 0.0081050 0.09003

Number of obs: 480, groups: fold, 10

Fixed effects:

Estimate Std. Error df t value Pr(>|t|)

(Intercept) 0.548270 0.035855 26.000000 15.291 1.67e-14 ***

model1 -0.012865 0.037482 446.000000 -0.343 0.731576

model3 0.354605 0.037482 446.000000 9.461 < 2e-16 ***

model4 0.310443 0.037482 446.000000 8.283 1.33e-15 ***

n_vv 0.100668 0.044012 45.400000 2.287 0.026888 *

voc 0.013286 0.007873 50.800000 1.687 0.097656 .

model1:n_vv -0.316526 0.053007 446.000000 -5.971 4.81e-09 ***

model3:n_vv -0.098109 0.053007 446.000000 -1.851 0.064848 .

model4:n_vv -0.096205 0.053007 446.000000 -1.815 0.070203 .

model1:voc -0.020092 0.009624 446.000000 -2.088 0.037395 *

model3:voc -0.010386 0.009624 446.000000 -1.079 0.281090

model4:voc -0.010831 0.009624 446.000000 -1.125 0.261038

n_vv:voc -0.038348 0.010141 119.600000 -3.782 0.000245 ***

model1:n_vv:voc 0.044586 0.013611 446.000000 3.276 0.001136 **

model3:n_vv:voc 0.015366 0.013611 446.000000 1.129 0.259543

model4:n_vv:voc 0.016682 0.013611 446.000000 1.226 0.220974

---

Signif. codes: 0 '***' 0.001 '**' 0.01 '*' 0.05 '.' 0.1 ' ' 1

Correlation matrix not shown by default, as p = 16 > 12.

Use print(x, correlation=TRUE) or

vcov(x) if you need it

**Statistical model for recall with the left model as right level, output from R:**

summary(model_random_intercept_rec, correlation=TRUE)

Linear mixed model fit by REML

t-tests use Satterthwaite approximations to degrees of freedom ['lmerMod']

Formula: rec ~ model * n_v * voc + (n_v * voc | fold)

Data: scores_models

REML criterion at convergence: -1161

Scaled residuals:

Min 1Q Median 3Q Max

-2.63530 -0.58922 -0.03702 0.55725 2.96797

Random effects:

Groups Name Variance Std.Dev. Corr

fold (Intercept) 8.523e-14 2.919e-07

n_vv 8.744e-14 2.957e-07 -1.00

voc 5.204e-15 7.214e-08 -1.00 1.00

n_vv:voc 3.670e-15 6.058e-08 0.98 -0.98 -0.99

Residual 4.088e-03 6.394e-02

Number of obs: 480, groups: fold, 10

Fixed effects:

Estimate Std. Error df t value Pr(>|t|)

(Intercept) -0.063141 0.018824 464.000000 -3.354 0.000861 ***

model1 0.032825 0.026621 464.000000 1.233 0.218176

model3 0.026540 0.026621 464.000000 0.997 0.319291

model4 0.296724 0.026621 464.000000 11.146 < 2e-16 ***

n_vv -0.050963 0.026621 464.000000 -1.914 0.056179 .

voc 0.051873 0.004833 464.000000 10.732 < 2e-16 ***

model1:n_vv 0.062812 0.037647 464.000000 1.668 0.095902 .

model3:n_vv -0.009013 0.037647 464.000000 -0.239 0.810892

model4:n_vv -0.305134 0.037647 464.000000 -8.105 4.88e-15 ***

model1:voc -0.030890 0.006836 464.000000 -4.519 7.89e-06 ***

model3:voc 0.066229 0.006836 464.000000 9.689 < 2e-16 ***

model4:voc 0.043178 0.006836 464.000000 6.317 6.27e-10 ***

n_vv:voc 0.035675 0.006836 464.000000 5.219 2.72e-07 ***

model1:n_vv:voc -0.035139 0.009667 464.000000 -3.635 0.000309 ***

model3:n_vv:voc -0.053777 0.009667 464.000000 -5.563 4.49e-08 ***

model4:n_vv:voc -0.025242 0.009667 464.000000 -2.611 0.009314 **

---

Signif. codes: 0 '***' 0.001 '**' 0.01 '*' 0.05 '.' 0.1 ' ' 1

Correlation matrix not shown by default, as p = 16 > 12.

Use print(x, correlation=TRUE) or

vcov(x) if you need it

**Statistical model for precision with the framing model as base level, output from R:**

summary(model_random_intercept_prec, correlation=TRUE)

Linear mixed model fit by REML

t-tests use Satterthwaite approximations to degrees of freedom ['lmerMod']

Formula: prec ~ model * n_v * voc + (n_v * voc | fold)

Data: scores_models

REML criterion at convergence: -820.3

Scaled residuals:

Min 1Q Median 3Q Max

-6.2026 -0.3867 0.0515 0.3777 5.1599

Random effects:

Groups Name Variance Std.Dev. Corr

fold (Intercept) 0.0058314 0.07636

n_vv 0.0053216 0.07295 -0.89

voc 0.0001568 0.01252 -0.99 0.81

n_vv:voc 0.0001020 0.01010 0.96 -0.98 -0.92

Residual 0.0081050 0.09003

Number of obs: 480, groups: fold, 10

Fixed effects:

Estimate Std. Error df t value Pr(>|t|)

(Intercept) 8.587e-01 3.585e-02 2.600e+01 23.950 < 2e-16 ***

model1 -3.233e-01 3.748e-02 4.460e+02 -8.626 < 2e-16 ***

model2 -3.104e-01 3.748e-02 4.460e+02 -8.283 1.33e-15 ***

model3 4.416e-02 3.748e-02 4.460e+02 1.178 0.2393

n_vv 4.463e-03 4.401e-02 4.540e+01 0.101 0.9197

voc 2.455e-03 7.873e-03 5.080e+01 0.312 0.7565

model1:n_vv -2.203e-01 5.301e-02 4.460e+02 -4.156 3.88e-05 ***

model2:n_vv 9.621e-02 5.301e-02 4.460e+02 1.815 0.0702 .

model3:n_vv -1.904e-03 5.301e-02 4.460e+02 -0.036 0.9714

model1:voc -9.262e-03 9.624e-03 4.460e+02 -0.962 0.3364

model2:voc 1.083e-02 9.624e-03 4.460e+02 1.125 0.2610

model3:voc 4.445e-04 9.624e-03 4.460e+02 0.046 0.9632

n_vv:voc -2.167e-02 1.014e-02 1.196e+02 -2.137 0.0347 *

model1:n_vv:voc 2.790e-02 1.361e-02 4.460e+02 2.050 0.0409 *

model2:n_vv:voc -1.668e-02 1.361e-02 4.460e+02 -1.226 0.2210

model3:n_vv:voc -1.317e-03 1.361e-02 4.460e+02 -0.097 0.9230

---

Signif. codes: 0 '***' 0.001 '**' 0.01 '*' 0.05 '.' 0.1 ' ' 1

Correlation matrix not shown by default, as p = 16 > 12.

Use print(x, correlation=TRUE) or

vcov(x) if you need it

**Statistical model for recall with the framing model as base level, output from R:**

summary(model_random_intercept_rec, correlation=TRUE)

Linear mixed model fit by REML

t-tests use Satterthwaite approximations to degrees of freedom ['lmerMod']

Formula: rec ~ model * n_v * voc + (n_v * voc | fold)

Data: scores_models

REML criterion at convergence: -1161

Scaled residuals:

Min 1Q Median 3Q Max

-2.63530 -0.58922 -0.03702 0.55725 2.96797

Random effects:

Groups Name Variance Std.Dev. Corr

fold (Intercept) 4.265e-12 2.065e-06

n_vv 1.059e-11 3.254e-06 -1.00

voc 2.078e-13 4.558e-07 -1.00 1.00

n_vv:voc 5.572e-13 7.464e-07 1.00 -1.00 -1.00

Residual 4.088e-03 6.394e-02

Number of obs: 480, groups: fold, 10

Fixed effects:

Estimate Std. Error df t value Pr(>|t|)

(Intercept) 0.233583 0.018824 464.000000 12.409 < 2e-16 ***

model1 -0.263899 0.026621 464.000000 -9.913 < 2e-16 ***

model2 -0.296724 0.026621 464.000000 -11.146 < 2e-16 ***

model3 -0.270184 0.026621 464.000000 -10.149 < 2e-16 ***

n_vv -0.356097 0.026621 464.000000 -13.377 < 2e-16 ***

voc 0.095051 0.004833 464.000000 19.665 < 2e-16 ***

model1:n_vv 0.367946 0.037647 464.000000 9.774 < 2e-16 ***

model2:n_vv 0.305134 0.037647 464.000000 8.105 4.88e-15 ***

model3:n_vv 0.296120 0.037647 464.000000 7.866 2.60e-14 ***

model1:voc -0.074068 0.006836 464.000000 -10.836 < 2e-16 ***

model2:voc -0.043178 0.006836 464.000000 -6.317 6.27e-10 ***

model3:voc 0.023051 0.006836 464.000000 3.372 0.000808 ***

n_vv:voc 0.010433 0.006836 464.000000 1.526 0.127616

model1:n_vv:voc -0.009896 0.009667 464.000000 -1.024 0.306486

model2:n_vv:voc 0.025242 0.009667 464.000000 2.611 0.009314 **

model3:n_vv:voc -0.028534 0.009667 464.000000 -2.952 0.003320 **

---

Signif. codes: 0 '***' 0.001 '**' 0.01 '*' 0.05 '.' 0.1 ' ' 1

Correlation matrix not shown by default, as p = 16 > 12.

Use print(x, correlation=TRUE) or

vcov(x) if you need it
